# Limb, sex, but not acute dietary capsaicin, modulate the near‐infrared spectroscopy‐vascular occlusion test estimate of muscle metabolism

**DOI:** 10.14814/phy2.15988

**Published:** 2024-03-27

**Authors:** Lauren M. Greaves, Kendall S. Zaleski, Alexs A. Matias, Abena O. Gyampo, Gaia Giuriato, Meaghan Lynch, Brian Lora, Tawn Tomasi, Emma Basso, Emma Finegan, Jack Schickler, Massimo Venturelli, Justin A. DeBlauw, Elena Shostak, Oliver E. Blum, Stephen J. Ives

**Affiliations:** ^1^ Health and Human Physiological Sciences Department Skidmore College Saratoga Springs New York USA; ^2^ Department of Kinesiology and Applied Physiology University of Delaware Newark Delaware USA; ^3^ Department of Neurosciences, Biomedicine and Movement Sciences University of Verona Verona Italy

**Keywords:** desaturation rate, mitochondria, muscle VO_2_, sex differences, slope 1, vascular occlusion test

## Abstract

The downward slope during the near‐infrared spectroscopy (NIRS)‐vascular occlusion test (NIRS‐VOT) is purported as a simplified estimate of metabolism. Whether or not the NIRS‐VOT exhibits sex‐ or limb‐specificity or may be acutely altered remains to be elucidated. Thus, we investigated if there is limb‐ or sex specificity in tissue desaturation rates (DeO_2_) during a NIRS‐VOT, and if acute dietary capsaicin may alter this estimate of muscle metabolism. Young healthy men (*n* = 25, 21 ± 4 years) and women (*n* = 20, 20 ± 1 years) ingested either placebo or capsaicin, in a counterbalanced, single‐blind, crossover design after which a simplified NIRS‐VOT was conducted to determine the DeO_2_ (%/s), as an estimate of oxidative muscle metabolism, in both the forearm (flexors) and thigh (vastus lateralis). There was a significant limb effect with the quadriceps having a greater DeO_2_ than the forearm (−2.31 ± 1.34 vs. −1.78 ± 1.22%/s, *p* = 0.007, *η*_*p*_^2^ = 0.19). There was a significant effect of sex on DeO_2_ (*p* = 0.005, *η*_*p*_^2^ = 0.203) with men exhibiting a lesser DeO_2_ than women (−1.73 ± 1.03 vs. −2.36 ± 1.32%/s, respectively). This manifested in significant interactions of limb*capsaicin (*p* = 0.001, *η*_*p*_^2^ = 0.26) as well as limb*capsaicin*sex on DeO_2_ (*p* = 0.013, *η*_*p*_^2^ = 0.16) being observed. Capsaicin does not clearly alter O_2_‐dependent muscle metabolism, but there was apparent limb and sex specificity, interacting with capsaicin in this NIRS‐derived assessment.

## 1 INTRODUCTION

Traditionally, whole‐body measures of metabolism have been achieved through direct or indirect calorimetry assessment of resting metabolic rate (RMR) (Divakaruni & Jastroch, 2022). While these are often considered the gold standard, and of great utility, they are whole‐body estimates or a conglomerate signal and thus lack specificity to individual muscle or muscle groups, a key contributor to metabolism and glucose homeostasis (Merz & Thurmond, 2020). Invasive biopsies of muscle tissue allow for ex vivo assessment of respiration via permeabilized muscle fiber or isolated mitochondrial preparations (Divakaruni & Jastroch, 2022; Park et al., 2014). Such a procedure is highly invasive for participants and not readily accessible to all researchers. Consequently, in vivo approaches such as magnetic resonance spectroscopy (MRS) avoids this invasiveness, by assessing local muscle changes in phosphorus metabolites and phosphocreatine (PCr) recovery kinetics, to then estimate muscle oxidative capacity with stimulation or contractions (mVO_2_) (Haseler et al., 1999; Layec et al., 2013; Richardson et al., 2001). However, such an approach comes at a high financial cost using equipment inaccessible to most researchers, and contraindications, for example, pacemakers, may limit its use in certain patient populations. Thus, feasible alternative assessments of muscle metabolism are needed.

A newer, non‐invasive, relatively cost‐effective technique has been proposed using near‐infrared spectroscopy (NIRS)‐derived measures of metabolic function in vivo, which has been validated against MRS (McCully et al., 1994; Ryan et al., 2013; Sumner et al., 2020). Due to its ease of applicability and non‐invasive nature, the technology surrounding NIRS has sparked increasing attention in its evaluation of regional circulation and metabolism (Barstow, 2019; Jones et al., 2016; Rogers et al., 2023; Scheeren et al., 2012). A prior study using NIRS found the recovery of O_2_Hb (oxygenated hemoglobin/myoglobin) and/or Hbdiff (O_2_Hb – HHb) were on par with recovery kinetics of PCr after exercise (McCully et al., 1994), suggesting that NIRS may be a novel approach to measuring kinetic changes in skeletal muscle oxygen consumption with contractions. Newer approaches now utilize arterial occlusions to non‐invasively isolate oxygen consumption from oxygen delivery (Azevedo et al., 2022; Ryan et al., 2013). However, given previously described sex differences in resting metabolic rate (RMR) (Arciero et al., 1993) and NIRS‐derived vascular reactivity (Keller et al., 2023; Rasica et al., 2022; Soares et al., 2018), including our group (Zaleski, Matias, et al., 2023) much less is known about sex differences in NIRS‐derived estimates of metabolism. This is especially true regarding the less complex NIRS‐vascular occlusion test (NIRS‐VOT) (McLay et al., 2016; Rogers et al., 2023). The NIRS‐VOT may simultaneously provide an estimate of resting muscle metabolism through a single vascular occlusion with minimal analysis of the downslope (slope 1 (McLay et al., 2016) or desaturation, DeO_2_) in S_t_O_2_ (Keller et al., 2023; Keller & Kennedy, 2021; Rogers et al., 2023). Though somewhat similar from prior methods, the NIRS‐VOT differs as it is an assessment of resting metabolism, likely through oxidative means, but perhaps through divergent bioenergetic pathways. Whether there is sex specificity in this assessment of metabolism is unresolved as the few prior studies using this approach have not controlled for the menstrual cycle phase (Keller et al., 2023; Keller & Kennedy, 2021; Rasica et al., 2022), which could influence metabolism (Benton et al., 2020). Further, there has been considerable research on metabolic and vascular differences between the upper and lower extremities (Richardson et al., 2006), but whether there is limb‐specificity in the DeO_2_ is unknown. Finally, it is not yet known whether the DeO_2_ is sensitive to acute alterations in metabolism.

Capsaicin, (8‐methyl‐N‐vanillyl‐trans‐6‐nonenamide), is a key component of spicy peppers, a known agonist for the transient receptor potential vanilloid channel‐1 (TRPV_1_), and dietary consumption may be associated with improved metabolism (Panchal et al., 2018; Wahlqvist & Wattanapenpaiboon, 2001; Zheng et al., 2017). Capsaicin has been documented to have general antioxidant, and anti‐inflammatory effects (Basith et al., 2016; Reyes‐Escogido et al., 2011), as well as specific effects on the autonomic nervous system, muscle, and the vasculature (Giuriato et al., 2022; Ives et al., 2017; Zaleski, Gyampo, et al., 2023). In terms of metabolism, a human trial administered a relatively high dose of capsaicin for 3 months demonstrated increased fat oxidation and elevated resting energy expenditure (REE) (Lejeune et al., 2003). Capsiate, a non‐pungent capsaicinoid is known to have similar metabolic effects (Inoue et al., 2007), and consuming non‐pungent peppers revealed an increase in VO_2_, even after a single acute dose in mice and humans (Ohnluki et al., 2001; Ohnuki et al., 2001). In humans, review of prior studies assessing long‐term intake of capsaicin identified elevated energy metabolism via VO_2_ (Ludy et al., 2012). Although the locus of such metabolic improvement cannot be ascertained in such models, these findings provide evidence that dietary capsaicin can alter metabolism, but muscle‐specific effects remain unknown. Few studies have investigated the use of acute capsaicin intake, and therefore, there is a need to understand how these metabolic mechanisms are affected by such supplementation. Future studies should use NIRS‐derived assessments, such as the NIRS‐VOT, to determine the effect of capsaicin on muscle‐specific oxidative metabolism, as this would also provide insight into the plasticity of this measurement.

Accordingly, the purpose of the present study was to determine the effects of acute dietary capsaicin treatment on resting muscle metabolism using StO_2_ desaturation rate (DeO_2_) during a NIRS‐VOT and to assess if there is limb or sex specificity in this assessment of metabolism. It was hypothesized that acute capsaicin treatment would promote greater desaturation rates suggestive of greater resting metabolism, but there may be limb or sex specificity basally, or in the response to acute dietary capsaicin.

## 2 METHODS

### 2.1 Subjects and general procedures

Forty‐five young, healthy participants (25 males, 20 females) aged 18–24 years old, were recruited for the present study. Healthy subjects were defined as individuals free of any renal, metabolic, cardiovascular, musculoskeletal, gastrointestinal, or neural diseases, which were determined using health history questionnaires. Furthermore, participants who were current or recent (less than 6 months) smokers or had any known sensitivity to spicy foods (i.e., hot peppers, jalapenos, paprika, etc.) or fiber (psyllium husk) were excluded from the study. Health histories and participant eligibility were reviewed on an individual basis. Physical activity was recorded using the International Physical Activity Questionnaire‐Short form (IPAQ‐SF) (Craig et al., 2003).

Participants were asked to refrain from ingesting any vitamins or ergogenic supplements (i.e., L‐Arginine, Citrulline‐Malate, Pre‐Workout) at least 48 h prior to each experimental visit, and to abstain from alcohol for 24 h, caffeine for 12 h, and having eaten for 4 h prior to testing. Volunteers were asked to follow a “low spicy food” diet, limit vigorous activity 24 h prior to their visit, and follow a similar sleep regimen throughout the duration of the study. For female participants, both experimental visits were completed during the first 7 days of their menstrual cycle, or during the placebo phase of oral contraceptives. This timeframe was chosen because it is when sex hormone levels are expected to be stable, at their lowest, and best reduce the acute impact of estrogen and other female sex hormones (Fu et al., 2009; Harris et al., 2012). Phase was determined via self‐report as in previous studies (Ives et al., 2011, 2013). Prior to participation in this study, written and informed consent was obtained from each subject. Approval for this study was granted by the Human Subjects Institutional Review Board of Skidmore College (IRB #1806‐728) and was conducted in accordance with the Declaration of Helsinki.

### 2.2 Procedures

The present study utilized a counterbalanced, single‐blind, placebo‐controlled, within‐subjects crossover design. This study aimed to focus on the potential metabolic consequences of capsaicin using the desaturation slope, but was part of another study that focused on the vascular reactivity which was published previously (Zaleski, Matias, et al., 2023). All subjects underwent three visits, including an initial screening and two experimental visits. During the screening visit, health histories and self‐reported physical activity assessments were obtained, and the participants' height and weight were collected using standard anthropometric techniques. Handgrip maximal voluntary contraction (MVC) was determined over three trials separated by 90 s, and the highest value recorded from the dynamometer (TSD121C, Biopac). If deemed eligible for the study, participants were enrolled and assigned, through counterbalance, to begin in one of two conditions: 2 × 90 mg of capsaicin capsules (Capsicool, Nature's way) or placebo control of 2 × 500 mg fiber capsules (Psyllium Husk, Kirkland Signature) sourced from a single batch. All capsules were taken orally 30 min prior to arrival. This timeframe was selected as the maximal concentration (*C*_max_) occurs within 60 min of administration (Suresh & Srinivasan, 2010). The fiber capsules were selected as they were of similar taste (encapsulated to maintain blinding) and appearance (e.g., size, color, texture, etc.) to the capsaicin capsules. In a recent study from our laboratory, these capsules were assessed for capsaicinoid content and found the total amount of capsaicin and dihydrocapsaicin to be 1.9 and 0.7 mg, respectively, in the experimental intervention, with the capsaicinoid content undetectable in the placebo (Zheng et al., 2017). This dose has been used in our prior studies and has been shown to elicit physiological changes such as sex‐specific alterations in cardiac autonomic nervous system activity, urinary levels of nitrite and nitrate, microvascular responsiveness, and attenuated peripheral fatigue development and late decreases in proinflammatory markers in males (Giuriato et al., 2022; Zaleski, Gyampo, et al., 2023; Zaleski, Matias, et al., 2023).

Upon arrival for the experimental visits, the participants were positioned supine and instrumented with a frequency‐domain multi‐distance near‐infrared spectroscopy (NIRS) system (Oxiplex TS, ISS) with light emitting and detecting sensor to assess tissue oxygen saturation to the vastus lateralis and wrist flexor muscle group (Rosenberry et al., 2018; Scheeren et al., 2012). Prior to placement, the probe was calibrated using a calibration block with known absorption and scattering characteristics. Before placement, the forearm (FA) and thigh were shaved, and cleaned with alcohol swabs, and double‐sided adhesive tape was applied to the probe. The NIRS probe was then positioned on the belly of the participants' right vastus lateralis quadriceps (Q) muscle, proximolateral to the knee, which was then covered and further secured with a bandage to minimize light exposure to the probe. Additionally, a rapid type vascular cuff (SC12D, 13 cm width) was positioned proximally above the probe on the vastus lateralis quadriceps muscle. To understand the potential gastrointestinal (GI) discomfort of the capsaicin tablets, a self‐reported measure was obtained using a 100 mm visual analogue scale (VAS), with the left anchor of “no discomfort” and right anchor of “severe discomfort”, similar to previous studies (Dudar et al., 2020). Participants were asked to lay quietly for 10 min in a temperature‐controlled dimly lit room.

**FIGURE 1:**
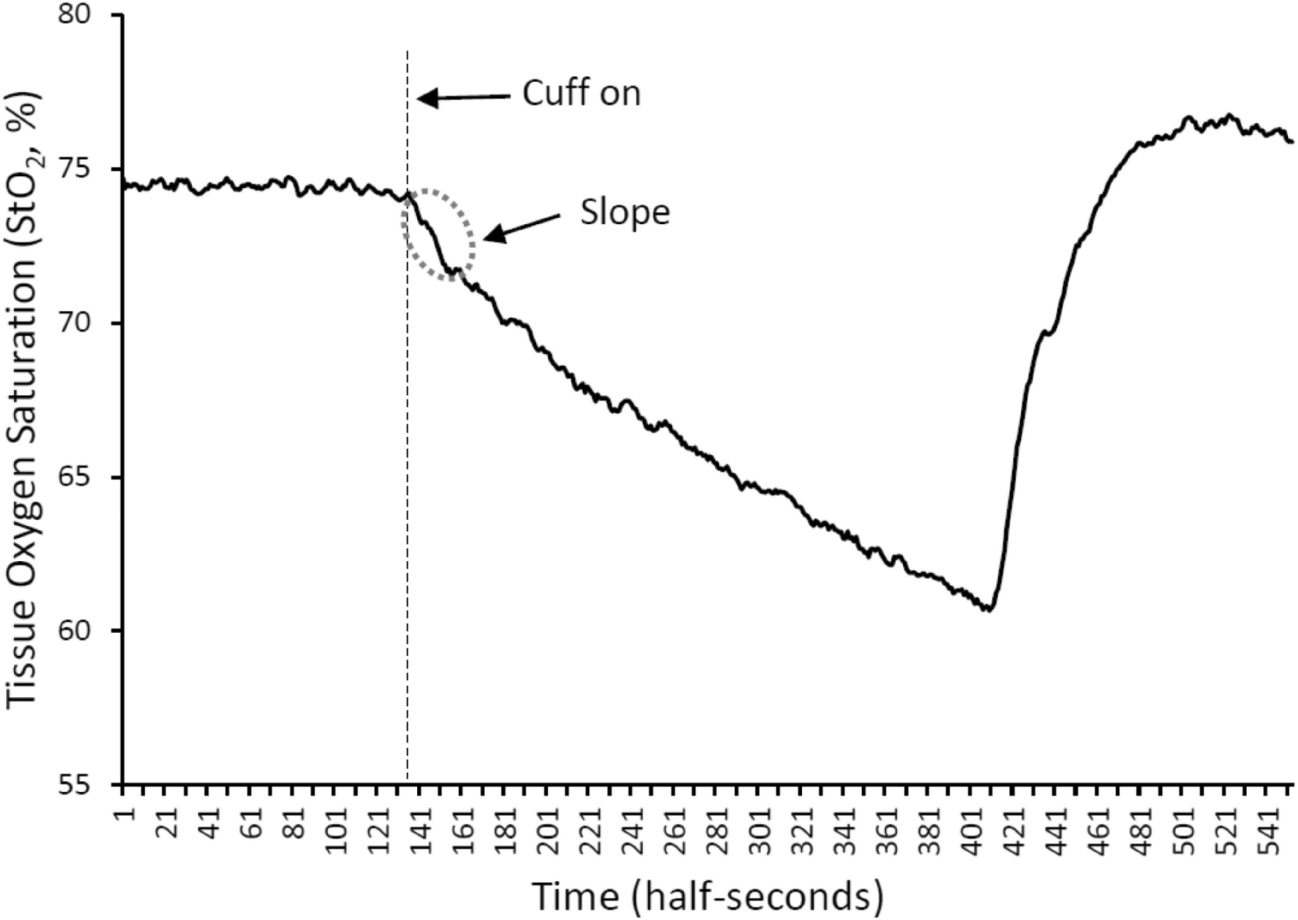
Representative tracing and overview of analysis for the desaturation slope (DeO_2_) using the tissue oxygen saturation (StO_2_). These data were acquired at 2 hz and thus each data point was acquired in half‐second intervals.

The NIRS‐VOT differs from the more complex approach pioneered by McCully and colleagues, in a number of ways, one of which is it requires 6 to 22+ repeated occlusion trials for curve fitting, correction for shifts in blood volume, and mathematical modeling to estimate VO_2_ (Azevedo et al., 2022; Ryan et al., 2013). Another couple of key differences are the physiological state, resting in the case of the NIRS‐VOT, versus post‐exercise/contractions in the McCully protocol, as well as the bioenergetic system (PCr shuttle in the recovery protocol vs. basal oxidative metabolism), although they likely share a common pathway of the mitochondria. The NIRS‐VOT mirrors the classic flow mediated dilation (FMD) ischemia reperfusion protocol (Ives et al., 2014). After rest, baseline tissue oxygen saturation (StO_2_, %) was assessed for 2 min as part of a standard NIRS‐vascular occlusion test (NIRS‐VOT) (Keller et al., 2023; Keller & Kennedy, 2021; Rogers et al., 2023). The vascular occlusion procedure was then conducted with the rapid cuff inflator inflated to supra systolic pressure of 220 mmHg using a rapid cuff inflator (E20, Hokanson, Bellevue, Washington) to elicit desaturation of the limb over a period of 5 min (Keller et al., 2023; Keller & Kennedy, 2021; Rogers et al., 2023). This pressure was chosen based on experience and prior research that has documented that arterial occlusion pressure in the leg, confirmed with ultrasound doppler, in supine position is ~150 mmHg (Loenneke et al., 2012; Sieljacks et al., 2018), and in the arm it is recommended for pressure to be systolic blood pressure +50 mmHg (Barstow, 2019), and using a wider cuff, as in the current study, requires ~50% lower pressure to achieve arterial occlusion (Weatherholt et al., 2019). The slope of change in the tissue oxygen saturation StO_2_ (%/s) was obtained in the first 10 s following cuff inflation (see Figure 1) to assess the limbs' desaturation slope (DeO_2_) and thus an estimate of the resting metabolic rate or oxygen utilization in skeletal muscle (Azevedo et al., 2022). This time point, and associated analysis, was chosen because it better reflects the more comprehensive NIRS‐based analysis of muscle VO_2_ that is often coupled with exercise (Azevedo et al., 2022; Ryan et al., 2013). The NIRS‐VOT test has been documented to have good to excellent test–retest reliability on slope 1 (desaturation) (Keller & Kennedy, 2021; Rogers et al., 2023). A single measurement was performed as in prior studies (Keller et al., 2023; Keller & Kennedy, 2021; Rogers et al., 2023). These procedures were repeated on the participants' right forearm with probe placement over the wrist flexor group muscle belly prominence with the rapid cuff inflator positioned above the probe but proximal to the antecubital space. Time between site measurements was about 20 min. Probe locations were marked with permanent marker for identical placement on the subsequent visit. Measurement of skinfold thickness was assessed using calipers (Lange, Beta Technology). To ensure adequate signal to muscle and determine the potential relation of measure to skinfold thickness, measurements were made between the emitter and detector at each measurement site in duplicate, and if values differed by more than 1 mm, a third measurement was taken, and values were then averaged (Barstow, 2019). Subjects returned to the lab for their second experimental visit, which was performed at least 48 h after the first test, at the same time of day as the first test, but no more than 1 week later.

### 2.3 Statistical analysis

Statistical analyses were conducted using open‐source software (JASP v0.18.0, University of Amsterdam, Netherlands). In the F test family, using standard parameters (e.g., alpha = 0.05, *f* = 0.25) for 2 groups and 4 measurements, in a repeated measures design, to achieve minimum statistical power of 0.8, a total sample of 24 subjects is required to detect a significant interaction effect (G*Power, v3.1, Kiel, Germany). Assessments of statistical significance differences by single effect, sex, or condition (placebo vs. capsaicin) were analyzed using independent and paired samples *t*‐tests, respectively. Comparisons of sex, condition, limb, and their potential interactions, were analyzed using a multivariate mixed model analysis of variance (ANOVA). Tests of normality were conducted, and if a significant violation was found, an appropriate correction was made to adjust the degrees of freedom. To understand the potential relations between subject characteristics (i.e., body mass index, maximal voluntary contraction, etc.) and DeO_2_ correlational analysis was performed. If assumptions of normality were violated, a non‐parametric alternative was applied (Spearman's Rho). Markers of effect size, appropriate for a given statistical test, are included to complement *p*‐values. Significance was established at *p <* 0.05. Data are presented as mean ± standard deviation (SD), unless noted otherwise.

## 3 RESULTS

### 3.1 Participant characteristics

The study included 45 participants, consisting of young healthy men (*n* = 25) and women (*n* = 20). Unsurprisingly, the females, on average, had significantly lower height (*p <* 0.001, *d =* 2.454) and weight (*p =* 0.003, *d =* 0.935) when compared with the males, but the sexes were otherwise well‐matched as assessed by body mass index (BMI, Table 1, *p* > 0.05). There were no other sex differences in the self‐reported physical activity levels on a minutely, hourly, or daily basis for moderate, mild, and sedentary behaviors (all *p >* 0.05, Table 1). Only in the days of vigorous physical activity each week, there was a significant sex difference such that the males performed vigorous activities more often each week than the females (*p =* 0.006, *d =* 0.869).

**TABLE 1:** Subject characteristics.

	Males (*n* = 25)	Females (*n* = 20)
Age (year)	21 ± 4	20 ± 1
Height (m)	1.77 ± 0.07	1.61 ± 0.06*
Weight (kg)	75.2 ± 10.9	63.60 ± 14.16*
BMI (kg/m^2^)	24.0 ± 2.8	24.4 ± 4.6
FA Skinfold Thickness (mm)	2.1 ± 0.7	2.8 ± 1.2
Q Skinfold Thickness (mm)	6.4 ± 2.6	6.5 ± 2.3
GID Placebo (VAS, cm)	0.4 ± 0.8	0.1 ± 0.3
GID Capsaicin (VAS, cm)	2.0 ± 1.9	2.1 ± 2.2
Vigorous PA (days/week)	3.7 ± 1.8	2.0 ± 2.2*
Vigorous PA (mins/day)	75.4 ± 51.0	52.5 ± 58.1
Moderate PA (days/week)	3.0 ± 2.4	2.3 ± 2.4
Moderate PA (mins/day)	70.2 ± 100.5	56.0 ± 70.2
Light PA (days/week)	5.9 ± 1.8	6.1 ± 1.00
Light PA (mins/day)	76.8 ± 81.8	67.6 ± 52.8
Sedentary Activity (hours/day)	6.9 ± 5.6	5.0 ± 2.7

Abbreviations: BMI, Body mass index; GID, gastrointestinal discomfort using a 100 mm; PA, physical activity; VAS, visual analog scale.

* *p <* 0.05 Males vs. Females. Data are means ± SD.

When assessing the gastrointestinal discomfort (GID) with the oral capsules, no sex differences were observed with the placebo (*p =* 0.16, *d =* 0.46) or capsaicin (*p =* 0.70, *d =* −0.12) capsules, however, there was a significant condition effect (*p <* 0.001), establishing that the capsaicin capsules produced greater GID than the placebo capsule, though the discomfort was still relatively mild (~2 out of 10 on the VAS, Table 1).

### 3.2 Sex‐, limb‐ and capsaicin‐induced differences in NIRS‐derived desaturation slope

**FIGURE 2:**
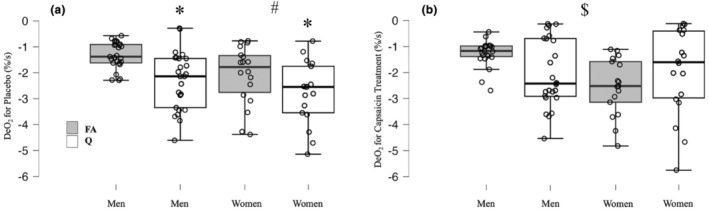
DeO_2_ in both the forearm flexor (FA ‐gray bars) muscles and the vastus lateralis of the quadriceps muscle (Q ‐white bars) of men (men =1, *n* = 25) and women (women =2, *n* = 20) under placebo (Panel a, left) and capsaicin conditions (Panel b, right). Data are presented as median, upper and lower quartiles, and upper and lower bounds, with individuals plots overlaid. *Main effect of limb (*p* = 0.007, *η*_*p*_^2^ = 0.19). #Main effect of sex (*p* = 0.005, *η*_*p*_^2^ = 0.203). $Significant interactions of limb*capsaicin (*p* = 0.001, *η*_*p*_^2^ = 0.26) as well as limb*capsaicin*sex on DeO_2_ (*p* = 0.013, *η*_*p*_^2^ = 0.16).

There was a significant interaction of limb and capsaicin on the desaturation slope (*p* = 0.001, *η*_*p*_^2^ = 0.259, Figure 2). There was no significant interaction of limb and sex on the desaturation slope, although there was a trend towards significance (*p* = 0.056, *η*_*p*_^2^ = 0.101, Figure 2). There was no interaction of capsaicin and sex on the desaturation slope (*p* = 0.67, *η*_*p*_^2^ = 0.005, Figure 2). There was a significant interaction of limb, capsaicin, and sex on the desaturation slope (*p* = 0.013, *η*_*p*_^2^ = 0.163, Figure 2). There was a significant limb effect with the quadriceps having a greater desaturation slope than the forearm (*p* = 0.007, *η*_*p*_^2^ = 0.191 Figure 2). Irrespective of sex, the quadriceps had a 51% greater slope than the forearm (−2.495 vs. −1.649%/s, respectively) under placebo conditions. There was no effect of capsaicin on the desaturation slope (*p* = 0.151, *η*_*p*_^2^ = 0.058, *d* = −0.241, Figure 2). There was a significant effect of sex on the desaturation slope (*p* = 0.005, *η*_*p*_^2^ = 0.203, *d* = 0.602, Figure 2). Between sexes, the average percent difference in the desaturation slope between the quadriceps and forearms were −19% and −44%, respectively, with males having a slower desaturation slope.

### 3.3 Relations between subject characteristics and NIRS‐derived desaturation slope

We sought to explore the potential relations between subject characteristics, namely BMI, weight (kg), skinfold thickness, and handgrip maximal voluntary contraction, and desaturation slope for the FA and Q. Under placebo conditions, we identified a significant positive moderate correlation between the O_2_ desaturation slope in the FA and handgrip maximal voluntary contraction (MVC) (*p* = 0.016, *r*_s_ = 0.37). There was a significant negative moderate correlation between the skinfold and the O_2_ desaturation rate in the FA (*p* = 0.024, *r*_s_ = −0.34). However, there were no significant correlations between O_2_ desaturation slope in both FA and Q with weight (*p >* 0.919, *r*_s_ <0.13), Skinfold Q (*p =* 0.427, *r*_s_ = 0.126) or BMI (*p* > 0.898, *r*_s_ < −0.269).

## 4 DISCUSSION

The current investigation aimed to determine limb‐specific and sex‐specific differences that exist in the desaturation slope (DeO_2_) of upper and lower limb muscles as part of the NIRS‐VOT estimate of muscle metabolism, the potential relations between anthropometric measures such as MVC, BMI and adipose tissue thickness and DeO_2_, and whether ingestion of acute dietary capsaicin ingestion enhances the DeO_2_. The novel findings from this study include: DeO_2_ is largely dependent upon sex and limb, with the magnitude of sex difference somewhat dependent upon the limb of interrogation, and the leg (v. lateralis) having larger DeO_2_ in both men and women. We did not observe a difference in response to acute dietary capsaicin in DeO_2_. While DeO_2_ was seemingly unrelated to body weight or BMI, in a largely normal weight population, skinfold thickness in the forearm and muscle strength were moderately related to DeO_2_ in the forearm only. The findings from the present study may be important when designing or interpreting studies using the NIRS‐VOT to assess oxidative muscle metabolism in young adult men and women. Further, the present study highlights that prior studies of capsaicin‐induced differences in whole‐body metabolism may depend on other tissues (e.g., adipose tissue lipolysis), and/or the effects of capsaicin may be specific to population or dose.

### 4.1 Sex‐specific differences in NIRS‐VOT desaturation slope estimate of muscle metabolism

Using whole‐body assessments of resting energy metabolism, or resting metabolic rate, previous researchers have identified that women, on average, have lower metabolic rates than men, although if demographic factors such as lean body mass are accounted for the differences are diminished (Arciero et al., 1993; Buchholz et al., 2001; Ferraro et al., 1992; Klausen et al., 1997). The NIRS‐VOT has been suggested to be a simplified approach for the simultaneous assessment of muscle metabolism (Keller & Kennedy, 2021; Rasica et al., 2022; Rogers et al., 2023). Assessing muscle metabolism has been demonstrated to be sex‐specific such that men desaturate quicker during a NIRS‐VOT than their female counterparts, suggesting greater muscle VO_2_ (Keller et al., 2023; Keller & Kennedy, 2021). In agreement, we also observed such sex specificity in the DeO_2_ in the present study; however, unlike prior studies by Keller and colleagues (Keller et al., 2023; Keller & Kennedy, 2021), the direction of the difference was reversed. Specifically, we report females having a higher desaturation slope than males, on average by 32%.

This discrepancy may be due to sex differences in tissue distribution, with fat‐free mass typically being higher in males compared to female counterparts as well as metabolic capacity, and resting metabolic rate (RMR) which may be responsible for the inherent sex differences identified previously (Arciero et al., 1993; Buchholz et al., 2001; Ferraro et al., 1992; Klausen et al., 1997). Females are also known to have a higher concentration and thicker adipose tissue layer underneath the skin and over muscle tissue (Azevedo et al., 2022). NIRS assessments have a penetration depth of approximately half the distance between the emitter and the received (Homma et al., 1996), thus subcutaneous adipose thickness could hypothetically explain, at least in part, the differences between sexes. Critically, we assessed skinfold thickness at both limb sites to determine if there were sex differences in subcutaneous adipose tissue thickness, and found that, in this sample, skinfold thickness was not higher in women compared to men. Interestingly, while one might suggest that a thicker subcutaneous adipose layer may compromise the mVO_2_, as assessed by the DeO_2_, in this case, the women had similar skinfold thickness, and further a greater DeO_2_ doesn't fit that hypothesis, given the dramatically lower metabolic capacity of adipose tissue when compared to skeletal muscle (Hoffmann et al., 2020). From a technical perspective, the emitter‐detector distance of the NIRS sensor used in the present study is 2–3.5 cm, suggesting, via the one‐half rule, that penetration depth is 1–1.75 cm, which exceeds the on‐average skinfold thicknesses of 2.4 and 6.3 mm in the forearm and v. lateralis. Although, we cannot exclude the potential contribution of subcutaneous adipose tissue metabolism. Indeed, a recent meta‐analysis of sex differences in mitochondrial metabolism indicates greater mitochondrial content in white adipose tissue of females (Junker et al., 2022), which could conceivably contribute to the, on average, greater DeO_2_ in women.

Though not assessed in the current study, muscle mass might provide some explanation of the basal sex or limb differences; although considering that StO_2_ is already a relative measure within a specified region of interest in muscle, and not the whole muscle itself, it is unclear if normalizing the measure would alter the kinetics but could be considered for future study. Finally, the men and women who participated in the current study were well‐matched on most parameters, including physical activity and BMI, which if not controlled for, along with the metabolic consequences of the menstrual cycle phase (Benton et al., 2020), in prior studies (Keller & Kennedy, 2021; Rasica et al., 2022) may contribute to the discrepancies in the directionality or magnitude of such sex differences.

Regarding potential cellular mechanisms at play, previous reports show mitochondrial function and the metabolic phenotype will also play a role in the differences between sex, such that anatomical location and fiber‐type composition can be sex‐dependent (Cardinale et al., 2018; Haizlip et al., 2015). Men display larger relative composition of fast muscle fibers (i.e., 66% of ATPase‐positive fibers & 64% ~68% of MyHC‐II); whereas females present with a greater prevalence of slower type fibers (type‐I and IIA) (Haizlip et al., 2015). The prevalence of slower‐twitch fibers allows for a higher oxidative capacity in the female population, highlighting likely sex‐based differences in oxidative muscle metabolism (Haizlip et al., 2015). Sex differences in substrate metabolism may also play a role in the present study's disparate DeO_2_ results (Lundsgaard & Kiens, 2014). Females are also known to have lower plasma hemoglobin levels compared with males (Joannides et al., 2002), possibly demanding greater microvascular blood flow to maintain O_2_ delivery (Nishiyama et al., 2008), which, if clamped with cuffing, could result in a more rapid decline in intramuscular tissue oxygenation. Other studies have shown that women have faster central O_2_ transport (*Q*), as well as faster peripheral and pulmonary O_2_ extraction dynamics as compared to men (Beltrame et al., 2017). These faster VO_2_ dynamics were presented with the same metabolic demand as their male counterparts (Beltrame et al., 2017). Relatedly, our prior work demonstrated that using NIRS, women had greater reperfusion slopes, suggesting enhanced microvascular kinetics (Zaleski, Matias, et al., 2023). The observed greater DeO_2_ in women might be accounted for, in part, by likely higher relative composition of slow‐twitch fibers, greater fat utilization, and/or faster O_2_ kinetics.

### 4.2 Limb‐specific differences in NIRS‐VOT desaturation slope estimate of muscle metabolism

Previous studies have suggested limb‐specific differences may be due to variance in muscle mass between limbs for both sexes, as females tend to have lower leg muscle mass in the quadriceps muscle relative to that of males, while forearm mass is more similar between sexes (Nishiyama et al., 2008). To our knowledge, this is the first study to directly investigate limb differences in NIRS‐VOT derived DeO_2_. The present data show that on average, the quadricep muscles had a greater DeO_2_ than the forearm suggesting a greater O_2_ utilization. Though muscle mass was not assessed in the current study, it could conceivably provide an explanation of the limb differences in S_t_O_2_. Differences in myoglobin content between limbs may also affect the minimum and maximum S_t_O_2_ values but should not affect change during occlusion as expressed by DeO_2_. Invasive techniques, such as muscle biopsy and respirometry, or proton (^1^H) resonance spectroscopy (MRS) could provide a more detailed analysis of the potential physiological mechanisms that may explain the limb‐specific differences.

### 4.3 The effect of capsaicin treatment on NIRS‐VOT estimate of muscle metabolism

Brown adipose tissue (BAT) is known to play a key role in cold‐induced non‐shivering thermogenesis to maintain body temperature and is often the target of obese and metabolic disorder therapies in humans (Yoneshiro & Saito, 2015). Capsaicin has been shown to affect energy expenditure by triggering BAT in the same way as low temperatures do via non‐shivering thermogenesis (Saito & Yoneshiro, 2013; Zheng et al., 2017). A prior study demonstrated that 8 weeks of a 9 mg dose of capsaicin could increase BAT activity and increase thermogenesis in healthy individuals (Nirengi et al., 2016). Other potential physiological mechanisms of metabolic effects of capsaicin include: increased lipid oxidation and adipogenesis inhibition, modulation of the gastrointestinal tract and gut‐microbiome function, suppression of appetite and increased satiety regulated through neural circuits in the hypothalamus (Zheng et al., 2017). Collectively, dietary capsaicin could be beneficial for weight management and perhaps improve metabolic function. Despite not identifying a significant effect of acute capsaicin ingestion on DeO_2_ in the present study, it is likely that long‐term dosing may be needed, or the loci of the metabolic effects are outside of the muscle, such as in identified BAT depots (Sacks & Symonds, 2013), and should be considered for future study.

### 4.4 Experimental considerations

The present study was not conducted without limitation or additional consideration. While the NIRS technique has been suggested to be a beneficial method for the examination of tissue oxygen saturation and/or desaturation it is unable to precisely quantify concentrations of oxygen‐dependent chromophores, namely the inability to differentiate between hemoglobin and myoglobin (Barstow, 2019). The study also intentionally focused on the NIRS‐VOT slope 1 assessment of muscle metabolism is also likely influenced or tainted by adipose tissue metabolism, perhaps leading to lower estimated rates of metabolism. We do not have in house reproducibility data yet on this technique, but surmise it would be good given prior investigations on this issue (Keller & Kennedy, 2021; Rogers et al., 2023). There may also be discrepancy on how or when to analyze the downward slope (slope 1), in that in the present study we opted to analyze the initial 10 s downslope while others assess a 120 s period between 30 and 150 s (Keller et al., 2023; Keller & Kennedy, 2021; Rogers et al., 2023), further work is needed to determine the optimal method. Somewhat relatedly, we did not assess the StO_2_min and whether this relates to the DeO_2_ and/or ex vivo measures of oxidative metabolism remains to be determined. Regarding capsaicin, we utilized an absolute dose of capsaicin, which we have demonstrated to elicit physiological responses in other parameters (Giuriato et al., 2022), and in a sex‐specific manner (Zaleski, Gyampo, et al., 2023; Zaleski, Matias, et al., 2023), but we are still uncertain about what the optimal dose or dosing strategy (absolute vs. relative) may be. Further, while we controlled for the menstrual cycle phase, future studies should explore how the hormonal fluctuations with the menstrual cycle, may influence the DeO_2_, and with regards to sex differences. While participant characteristics did not differ at baseline in the present study, we did only assess a young, healthy, and relatively lean population. Future studies should investigate the DeO_2_ of clinically relevant populations through NIRS basally and in response to acute oral capsaicin.

## 5 CONCLUSIONS

The purpose of the present study was to determine the effects of acute dietary capsaicin treatment on muscle metabolism using NIRS StO_2_ desaturation rates (DeO_2_) and to assess limb or sex specificity in this assessment. In conclusion, acute capsaicin treatment does not appear to induce favorable changes in DeO_2_ in young healthy men and women. However, DeO_2_ is largely dependent upon sex and limb, with women, and the leg (v. lateralis) having larger DeO_2_, respectively. DeO_2_ does not appear to be related to body weight or BMI, but skinfold thickness and muscle strength were related to DeO_2_ in the forearm only. The findings from the present study may be important when designing or interpreting studies using the NIRS‐VOT technique to assess muscle metabolism (“slope 1”) in men and women, but exploration of the mechanisms involved is warranted.

## FUNDING INFORMATION

The American Heart Association has provided support to SJI (https://doi.org/10.58275/AHA.24AIREA1247045.pc.gr.189804).

## CONFLICT OF INTEREST STATEMENT

The authors have no competing interests to report.

## ETHICS STATEMENT

This study was reviewed and approved by the Human Subjects Institutional Review Board of Skidmore College (IRB #1806‐728) and was conducted in accordance with the Declaration of Helsinki. All participants provided written informed consent prior to participation.
